# Revealing protein functions based on relationships of interacting proteins and GO terms

**DOI:** 10.1186/s13326-017-0139-8

**Published:** 2017-09-20

**Authors:** Zhixia Teng, Maozu Guo, Xiaoyan Liu, Zhen Tian, Kai Che

**Affiliations:** 1 Department of Information Management and Information System, Northeast Forestry University, Harbin, 150040 China; 2 Department of Computer Science and Engineering, Harbin Institute of Technology, Harbin, 150001 China

**Keywords:** Protein function, Interacting protein, Gene ontology, Directed network

## Abstract

**Background:**

In recent years, numerous computational methods predicted protein function based on the protein-protein interaction (PPI) network. These methods supposed that two proteins share the same function if they interact with each other. However, it is reported by recent studies that the functions of two interacting proteins may be just related. It will mislead the prediction of protein function. Therefore, there is a need for investigating the functional relationship between interacting proteins.

**Results:**

In this paper, the functional relationship between interacting proteins is studied and a novel method, called as GoDIN, is advanced to annotate functions of interacting proteins in Gene Ontology (GO) context. It is assumed that the functional difference between interacting proteins can be expressed by semantic difference between GO term and its relatives. Thus, the method uses GO term and its relatives to annotate the interacting proteins separately according to their functional roles in the PPI network. The method is validated by a series of experiments and compared with the concerned method. The experimental results confirm the assumption and suggest that GoDIN is effective on predicting functions of protein.

**Conclusions:**

This study demonstrates that: (1) interacting proteins are not equal in the PPI network, and their function may be same or similar, or just related; (2) functional difference between interacting proteins can be measured by their degrees in the PPI network; (3) functional relationship between interacting proteins can be expressed by relationship between GO term and its relatives.

## Background

Characterizing protein functions is critical to understanding biological pathway, investigating disease and developing drugs [1, 2]. To elucidate protein functions, numerous research efforts have been made based on techniques ranging from sequence homology detection to text mining of scientific literature. However, only some of proteins are annotated with functional information for well-studied model organisms so far. The situations would be even worse for the other organisms.

Recently, biological network provides chance of studying gene and its products (e.g protein, microRNA) at system level [3, 4]. It is widely recognized that a protein performs functions according to its partners in protein-protein interaction (PPI) network. This recognition has motivated the development of numerous network-based methods for predicting protein function. These methods are proposed on the principle of guilt-by-association (GBA), that is, the closer the two proteins are in the network the more similar are their functions [5]. These network-based methods can be roughly grouped into two major classes: direct annotation methods [6–11] and model-assisted methods [12–15]. The comprehensive reviews of these methods can be found in [5, 16]. The direct annotation methods suppose that the interacting proteins share the same function and inferred protein functions by means of propagating the known functional annotations of its neighbors along the network edges. The model-assisted methods assume that proteins in the same group perform the same function. They firstly identify functional groups of proteins, and then annotate each group with the known functional annotations of the group’s members. In recent years, chi et al. [17] proposed a method named CIA, which iteratively updated annotations of a protein according to functional similarity between the protein and its partners. Wang [18] put forward a method named FCML to predict protein function by multi-label learning. The FCML took functional association between Gene Ontology (GO) terms [19] under consideration when it worked. Almost all of these methods predicted protein functions using PPI network and GO terms. In these methods, predicting protein function is to associate term with protein according to functional semantic information of the term. The result of predicting is named as annotation of proteins and an annotation is represented by a term. These methods have promoted the development of the protein functional predicting. However, most of them ignored some crucial information which affect the quality of prediction:

1. The PPI network is usually supposed as non-directional. In fact, it is commonplace in the PPI network that regulation relationship, upstream-downstream relations between interacting proteins when they are involved in signal transduction, transcriptional regulation, cell cycle or metabolism [20]. Moreover, it is reported by recent studies [21–23] that GBA is the exception rather than the rule in the PPI network and protein functions are determined by specific and critical interactions. Hence the relationship between interacting proteins may affect their functions and should be considered in the process of predicting protein functions.
2. In GO context, a series of standard terms are defined to describe characteristics of gene products (i.e. protein), and the terms are arranged as directed acyclic graph (DAG) hierarchy according to functional associations of them. Therefore, the functional information is not only expressed by semantics of terms but also contained in the hierarchy. Thus, the predictions of protein functions may be misled if the functional associations of terms are ignored. In fact, the information underlying in GO hierarchy are crucial for functional predicting of proteins.

In this paper, we mainly study two problems: (1) how to measure the functional difference between interacting proteins; (2) how to demonstrate functional difference between the interacting proteins in GO context. To solve above problems, we advance a novel method to predict protein functions by diffusing GO terms in the directed PPI network (GoDIN). Firstly, the relationship between interacting proteins is generalized as functional proactive-reactive. It is assumed that the proactive protein performs fewer and more specific functions than the reactive protein. And then a directed PPI network is generated according to the functional proactive-reactive relationships of interacting proteins. Secondly, a coefficient variation is defined to measure functional difference between interacting proteins. Finally, functional associations of GO terms are taken into consideration in the process of annotating interacting proteins. By a proposed iterative algorithm, GO terms are allocated to describe protein functions in the PPI network under the control of coefficient variations. The method will be illustrated in the following section.

## Methods

### Functional relationship between interacting proteins

As reported, many proteins play functional roles that are different from their neighbors in the PPI network. For example, a protein annotated with terms: “RNA transport”, “RNA binding” may involve in translation mechanism and bind with diverse functional unrelated proteins [23]. For instance, the function of proteins which help others fold correctly may be unrelated to that of their partners. These proteins are more likely to be hubs than others in the PPI network. The hubs often have many partners and may involve in several different biological activities. In general, a protein is multi-functional if it takes part in many different biological activities. As reported [22], the more multi-functionality of a protein is, the less specific is its function. Besides, Gillis et al. also found that the multi-functionality of a protein is highly correlated with its degree in the PPI network. Specifically, a protein with high degree may perform general function so that they could collaborate with other proteins in diverse biological activities. It can be considered that the low degree proteins are proactive and the high degree proteins are reactive in biological activities. Thus, the relationship between interacting proteins can be generalized as functional proactive-reactive according to their degrees in PPI network.

**Fig. 1:**
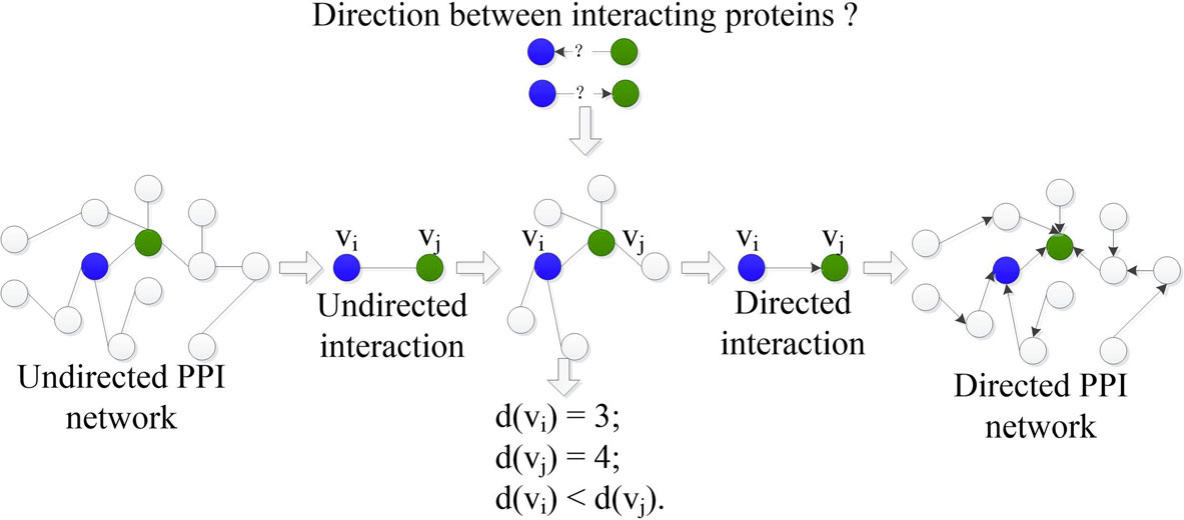
A simplified example of generating directed PPI network

Let the PPI network be generalized as a digraph, in which a node presents a protein and an arch links two interacting proteins, oriented from the low degree one to the high degree one. Note that, two interacting proteins with equal degree are linked with a bidirectional arch. Accordingly, as displayed in Fig. 1, a novel directed PPI network is generated from the original undirected PPI network.

As discussed above, the functional specificity may descent on the direction from proactive protein to reactive protein. Thus, the descent direction of functional specificity between two interacting proteins is defined as (1). In the formula, O(v_i_, v_j_) represents the descent direction of functional specificity between the two interacting proteins v_i_ and v_j_; d(.) denotes the degree of a protein in the PPI network. The formula means that: v_i_ plays more specific functions than v_j_ if O(v_i_, v_j_) =1; v_i_ play general functions than v_j_ if O(v_i_, v_j_) = −1; v_i_ and v_j_ are equal in the network and they share the same function if O(v_i_, v_j_) =0.

1 $$ O\left({v}_i,{v}_j\right)=\left\{\begin{array}{l}1,d\left({v}_i\right)<d\left({v}_j\right);\\ {}0,d\left({v}_i\right)=d\left({v}_j\right);\\ {}-1,d\left({v}_i\right)>d\left({v}_j\right).\end{array}\right. $$

### Measuring functional difference between interacting proteins

Here the functional difference between two interacting proteins is measured. It is considered that a protein perform specific functions if the protein is involved in few activities, vice versa. In the PPI network, the number of connections of a protein can reflect the number of activities the protein involves in. Thus, the functional specificity of a protein can be measured by degree of the protein in the PPI network. For two interacting proteins, their functional difference may be determined by the specificity difference of the functions which are performed by their interaction. Accordingly, a coefficient variation is defined to measure the functional difference between two interacting proteins. The functional coefficient variation between two interacting proteins v_i_ and v_j_ is marked as CV(v_i_, v_j_) and can be measured by (2).

2 $$ CV\left({v}_i,{v}_j\right)=\left|\frac{1}{d\left({v}_i\right)}-\frac{1}{d\left({v}_j\right)}\right| $$

### Annotate the interacting proteins with GO terms based on their functional difference

In traditional methods, the known GO terms of a protein were directly associated with interacting partners of the protein. These methods ignored the functional difference between the interacting proteins. In fact, the functions of interacting proteins may be same or similar, or related but different. Therefore, the relatives of known terms of a protein are selected to annotate interacting partners of the protein in our method.

To select relatives, it is supposed that an ideal term can annotate functions of neighbors exactly. Semantic value of the ideal term can be estimated based on those of the known terms of interacting proteins and functional coefficient variation and descent direction of functional specificity between them. In our method, semantic value of a term g_m_ is marked as S(g_m_) and computed by (3). In the formula, dep(g_m_) is the depth of g_m_, and desc(g_m_) is number of descendants of g_m_, and G_total_ is the total number of terms in GO hierarchy. Equation (3) is proved to be effective on calculating semantic values of terms in [24]. The semantic value of a term is big if the term has few descendants or lies at deep level in GO hierarchy. The bigger the semantic value of the term is, the more specific is the function described by the term.

3 $$ S\left({g}_m\right)= dep\left({g}_m\right).\left(1-\frac{\log \left( desc\left({g}_m\right)+1\right)}{\log \left({G}_{total}\right)}\right) $$

For interacting proteins v_i_ and v_j_, g_m_ is a known term of the protein v_i_; an ideal term g_m_^*^ can be inferred from g_m_ to annotate protein v_j_; and semantic value of g_m_^*^, S(g_m_^*^) can be computed by (4). Equation (4) is applicable for propagating terms between interacting proteins no matter which one of them is annotated.

4 $$ S\left({g}_m^{\ast}\right)={\left(1+ CV\left({v}_i,{v}_j\right)\right)}^{-O\left({v}_i,{v}_j\right)}.S\left({g}_m\right) $$

Based on semantic value of the ideal term, one or more relatives of the known term are selected to annotate protein v_j_ by (5). In the formula, R(g_m_) represents the set of relatives of g_m_ and g_m_^r^ is a relative of g_m_. To do this, the relatives of the known term, which are the most similar with the ideal term in term of the semantic value, are selected to describe functions of the protein v_j_. In practice, the selected relatives may be grandparents, parents, siblings, children or grandchildren of the known terms.

5 $$ \underset{g_m^r\in R\left({g}_m\right)}{\arg \min}\left|S\left({g}_m^r\right)-S\left({g}_m^{\ast}\right)\right| $$

Generally speaking, this process provides three kinds of predictions: (1) some ancestors of the known terms of the proactive protein may be appropriate to describe the reactive protein; (2) some descendants of the known terms of the reactive protein can annotate the proactive protein; (3) terms of two interacting proteins can be shared directly by them if the proteins are equal in the PPI network.

### Diffusing functional information in the PPI network

**Fig. 2:**
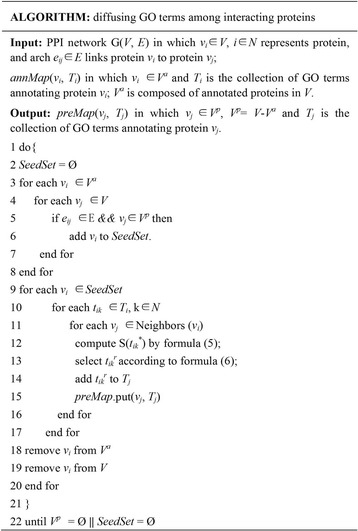
Algorithm for diffusing GO terms in the whole PPI network

To mine functional information as much as possible, an iterative algorithm is designed to diffuse GO terms in the whole PPI network. As described in Fig. 2, the algorithm includes four steps as following.

Step 1: Select seed proteins from annotated proteins of which proactive partners have not been annotated yet;

Step 2: Select relatives of known terms of seed proteins to describe functions of their interacting partners according to formulas (4) and (5);

Step 3: Update terms of seed proteins based on their annotated reactive partners according to formulas (4) and (5);

Step 4: Remove seed proteins from the annotated proteins; the edges related to the seed proteins cannot mediate diffusing between interacting proteins; and go to step 1 until all proteins in the PPI network are annotated or there does not exist annotated partners for remained unannotated proteins.

### Time complexity analysis

Given a PPI network including n proteins, the time complexity of determining functional relationship between proteins is O(n^2^). Similarly, the time complexity of measuring functional difference between proteins is O(n^2^) too. If the proteins is at most annotated by p GO terms, and the maximum degree of the proteins is k, the time complexity of diffusing functional information between two proteins is O(p × k). Accordingly, diffusing functional information in the whole PPI network is O(m × p × k) if there are m proteins are annotated in the PPI network. Based on these analysis, the time complexity of the GoDIN should be O(n^2^) + O(n^2^) + O(m × p × k). Because the maximal value of m is n and the maximal value of k is n-1, the time complexity of the GoDIN is about O(n^2^).

### A simple example of GoDIN

**Fig. 3:**
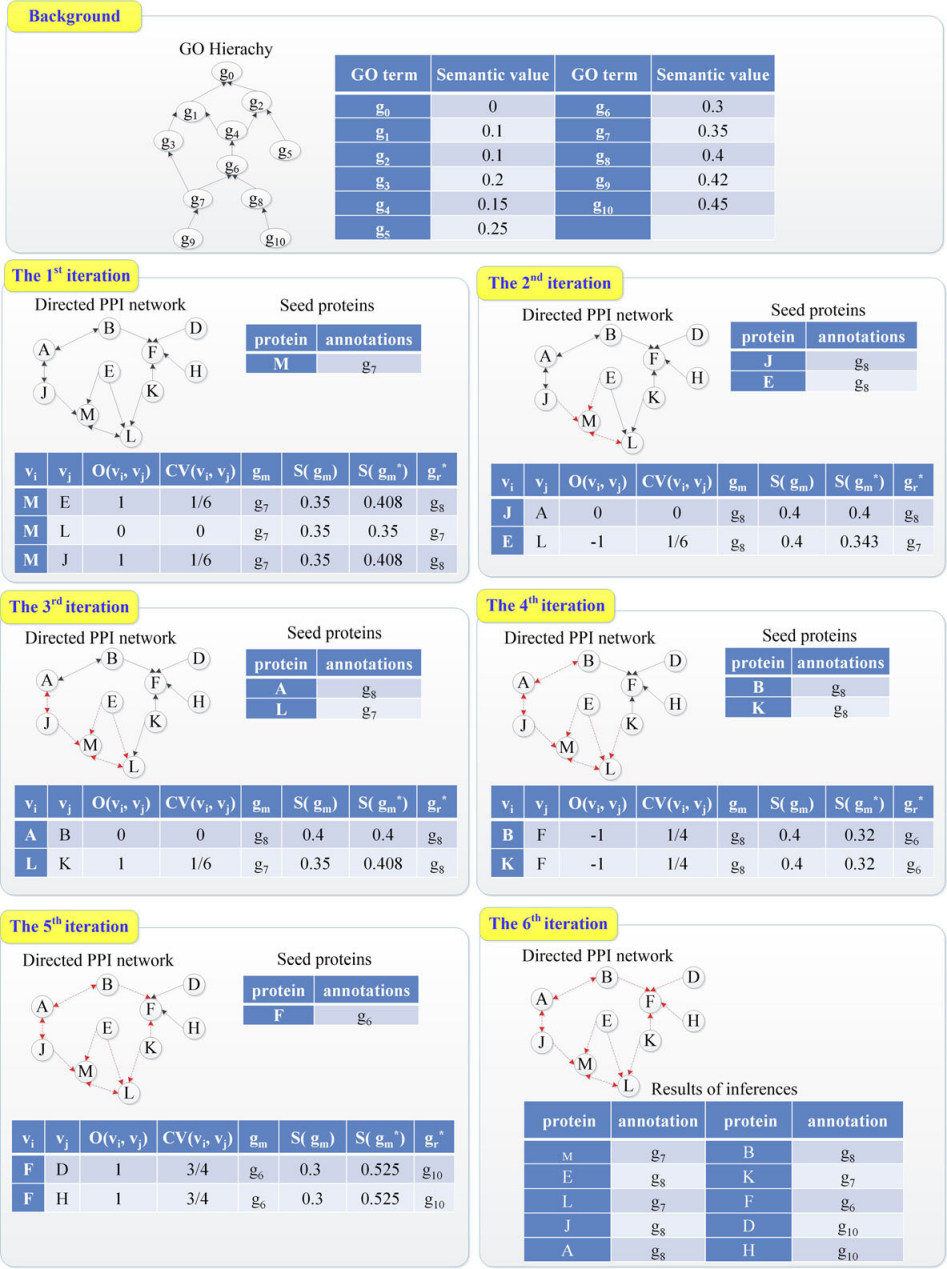
A simple example of how to diffusing GO terms though directed PPI network

To make our method clearly, a simple example is illustrated in Fig. 3. In the *Background* of the Fig. 3, some GO terms are organized as DAG, in which terms are linked with arches oriented from child to parent. As well, semantic values of the terms are all listed in the *Background*. Initially, protein M is annotated with term g_7_, L is annotated with term g_4_, and the other proteins in the subnetwork are unannotated. The functional proactive-reactive relationship between interacting proteins has been marked by an arch oriented from the proactive protein to the reactive protein. To reveal functions of the other proteins, annotations of M and L are diffused between interacting proteins iteratively. In our example, the diffusing process is finished through six iterations. In each round, some functional inferences are made and key information of inferences is displayed in a table. The key information include the descent direction of functional specificity between two interacting proteins (O(v_i_, v_j_)), functional coefficient variant between seed protein and its neighbor (CV(v_i_, v_j_)), known term (g_m_) and its semantic value (S(g_m_)), semantic value of ideal term (S(g_m_^*^)) and selected relative of the known term (g_r_^*^).

In the first iteration, M is regarded as a seed protein and its neighbors include E, L and J. According to the formula (1) and (2), O(M, E) is 1 and CV(M, E) is 1/6. The known GO term of M is g_7_ and the semantic value of g_7_, S(g_7_) is 0.35. By replacing parameters in the formula (4) with these data, S(g_7_^*^) is estimated as 0.408. According to the formula (5), g_8_ is appropriate to annotate protein E. Similarly, the annotations of L and J are predicted by the same means. Note that, because L has been annotated before diffusion, L’s term g_4_ should also be diffused to the seed proteins M. According to True Path Rule (TPR), g_4_ also annotates M if g_4_ is an ancestor of g_7_. Thus, the annotations of M cannot be changed by GO term g_4_. In addition, the protein M cannot be selected as a seed protein again and arches M ← J, M ← E, M↔L cannot be used to diffuse GO terms again.

In the second iteration, J, E and L are candidates for seed proteins. Because the protein L has a proactive annotated partner E, L cannot be taken as a seed protein. Therefore, J and E are selected as seed proteins. According to the formula (1), O(J, A) is 0, which means that the protein J and A share the same function. Thus, protein A can be annotated with term g_8_, which is also can be inferred though the formula (4) and (5). Different from L, A has not been annotated at all before diffusion, so it does not need to infer annotations of J from those of A. As for the seed protein E and its partner L, O(A, L) is −1 and CV(A, L) = 1/6 in term of the formula (1) and (2). Based on these parameters and S(g_8_), S(g_8_^*^) = (1 + 1/6)-1 × 0.4 = 0.343. Therefore, term g_7_ is selected to annotate L in term of the formula (5). After that, protein J and E cannot be regarded as seed proteins and arches J↔A and E → L cannot be used in the other iterations.

The processes of the 3rd, 4th, 5th iterations are similar to the previous iterations. Due to Space Limitations, the details of these iterations are not described here. In the 6th iteration, it can be found that all proteins in the subnetwork have been annotated already and no arch which can mediate diffusing between interacting proteins remains. Thus, the iteration is terminated and the diffusing of GO terms though the subnetwork is finished. The result of inferences are collected and listed in the table.

## Experiments and discussions

### Experimental datasets

Three high reliable PPI networks of saccharoinyces cerevisiae (Krogan, DIP, BioGRID) are used to study the performance of the proposed method GoDIN. Krogan [25] consists of interactions with probabilities above 0.273. The latest version of DIP was downloaded from database of interacting protein (http://dip.doe-mbi.ucla.edu/dip/) [26] on July 7, 2013. BioGRID consists of the physical interactions of saccharoinyces cerevisiae and it was downloaded from biological general repository for interaction datasets (http://thebiogrid.org/download.php) [27] on March 10, 2015. At the same time, the functional annotations of proteins of saccharoinyces cerevisiae were download from GO website and the annotations with evidence code ‘IEA’ (Inferred from Electronic Annotation), ‘NR’ (Not Recorded), ‘ND’ (No biological Data available), or ‘IC’ (Inferred by Curator) were excluded. The basic information of the three PPI networks are listed in Table 1. In the table, #PPI is the number of interactions in the network; #Proteins is the number of proteins in the network; #Annotated proteins is the number of the proteins with GO annotations; MF, BP and CC represent the annotation aspects: molecular function, biological process and cellular component respectively.

**Table 1:** Basic information of the three PPI networks

Network	#PPI	#Proteins	#Annotated proteins		
			*MF*	*BP*	*CC*
Krogan	7123	2708	2109	2424	2570
DIP	22,613	5097	3415	3941	4207
BioGRID	59,748	5640	4106	4754	5100

### Performance measures

Three widely-used measures: precision (P), recall (R) and f-measure (F) are employed to measure performance of GoDIN and other related methods. The measures are consistent with the famous Critical Assessment of Functional Annotations (CAFA) experiments [28]. P is the average precision of predictions about proteins on which at least one prediction was made. R is average recall of predictions on all target proteins. F is a harmonic mean between P and R, which gives an intuitive number for comparisons of the concerned methods. Supposed that x represents a target protein and K (x) is a set of known terms of x, P can be calculated as Eq. (6). In Eq. (6), P(x) is the set of predictive annotations; S is the target protein set for testing; m is the number of proteins which at least have one predictive term. Similarly, R and F can be computed by Eq. (7) and Eq. (8) respectively.

6 $$ P=\frac{1}{\mathrm{m}}\sum_{x\in S}\frac{\left|K(x)\cap P(x)\right|}{\left|P(x)\right|} $$

7 $$ R=\frac{1}{\left|\mathrm{S}\right|}\sum_{x\in S}\frac{\left|K(x)\cap P(x)\right|}{\left|K(x)\right|} $$

8 $$ \mathrm{F}=\frac{2P.R}{P+R} $$

### Functional relationship between interacting proteins

To study functional relationships of interacting proteins, the interactions of Krogan, DIP and BioGRID are analyzed thoroughly. Firstly, annotations of proteins in the networks are processed and the terms with evidence code ‘IPI’ (Inferred from Physical Interaction), ‘IGI’ (Inferred from Genetic Interaction) are excluded to avoid circular judgement. Secondly, the interactions are composed of two annotated proteins are selected for analysis. Finally, the selected interactions are grouped into: (1) the same annotation group, (2) the similar annotation group and (3) the related annotation group. The same annotation group consists of interactions which are composed of proteins with the same term. The similar annotation group consists of interactions which are composed of proteins with different terms of the same sub-ontology. Usually, the terms of the same sub-ontology are similar. The related annotation group consists of interactions which are composed of proteins only with terms of different sub-ontologies. The results of analysis are displayed in Table 2. In the table, #PPI^t^ is the number of interactions in the network; #PPI is the number of interactions in the group; Pct(%) presents the percentage of interactions in the group.

**Table 2:** Functional relationship of interacting proteins

Network	#PPI^t^	Same annotation		Similar annotation		#Related annotation	
		*#PPI*	*Pct(%)*	*#PPI*	*Pct(%)*	*#PPI*	*Pct(%)*
Krogan	6931	4114	59.36	2794	40.31	23	0.33
DIP	20,050	9816	48.96	10,182	50.78	52	0.26
BioGRID	58,765	30,154	51.31	28,464	48.44	147	0.25

From Table 2, it can be seen that nearly 60% of interactions in the three networks belong to the first group; about 40% of interactions belong to the second group; only less than 1% of interactions belong to the third group. As far as we know, none of methods relying on PPI network could annotate the interacting protein correctly in the third group. The traditional methods supposed that the interacting proteins share the same term. Thus, about 40% of functional predictions may not be correct. Meanwhile, the results suggest that the majority of interacting proteins share the same or similar terms, which is consistent with basic assumptions of GoDIN.

### Functional difference between interacting proteins

To investigate the influence of the degree on functional difference between proteins, the annotations and degrees of interacting proteins are analyzed. The analysis is performed on Krogan, DIP, BioGRID and the interactions of the networks are grouped into: the same annotation group and the similar annotation group. The former consists of interactions which are composed of proteins with the same term, and the latter includes interactions of which the proteins are annotated by similar terms. The results derived from the same annotation groups and the similar annotation groups are illustrated in Table 3 and Table 4 separately. In the tables, #PPI is the number of interactions in the group; #SameDeg is the number of interactions in which the interacting proteins with the same degree in the group; #DiffDeg is the number of interactions in which the interacting proteins with different degrees in the group; Pct(%) presents the percentage of interactions in the group. The results suggest that the majority of the interacting proteins have different degrees in the three networks. Some of the interacting proteins with different degrees are annotated by similar terms while the others share the same term.

**Table 3:** Relationship between function and degree of interacting proteins in the same annotation group

Network	#PPI	#SameDeg	Pct(%)	#DiffDeg	Pct(%)
Krogan	4114	243	5.9	3871	94.1
DIP	9816	531	5.4	9285	94.6
BioGRID	30,154	406	1.3	29,748	98.7

**Table 4:** Relationship between function and degree of interacting proteins in the similar annotation group

Network	#PPI	#SameDeg	Pct(%)	#DiffDeg	Pct(%)
Krogan	2794	91	3.26	2703	96.74
DIP	10,182	159	1.56	10,023	98.44
BioGRID	28,464	188	0.6	28,276	99.4

To explain this phenomenon, coefficient variation is used to measure functional difference between the interacting proteins. The coefficient variations of proteins with different degrees in the same annotation group are compared with those in the similar group. As shown in Fig. 3, the box-whisker plots are used to display the distributions of coefficient variations of different groups. In the figure, the distributions of the coefficient variations in the same annotation groups are represented by dashed boxes and lines. Meanwhile, the distributions of coefficient variations in the similar annotation groups are represented by solid boxes and lines. As known, the bottom and top of the boxes are always the first and third quartiles of coefficient variations, and the bands inside the boxes are the second quartiles (the median) of coefficient variations, and the hollow spots inside the boxes are the averages of coefficient variations. For clear, the same annotation groups of the three networks: Krogan, DIP and BioGRID are marked as SameKrogan, SameDIP, SameBIO respectively. Accordingly, the similar annotation groups of those networks are signed as SimilarKrogan, SimilarDIP and SimilarBIO.

**Fig. 4:**
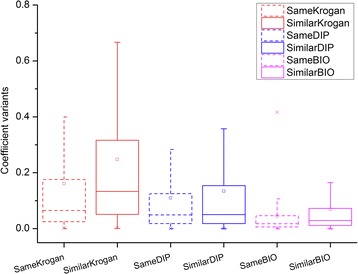
Comparison of coefficient variants based on different annotation groups

As displayed in Fig. 4, coefficient variations in the two different groups of the same network show obvious different distributions. According to the median, average, the first and third quartiles, the coefficient variations in the same annotation groups are higher than those in the similar annotation groups. This may suggest that the functional differences of interacting proteins in the same annotation groups are smaller than those in the similar annotation groups. Sometimes, although the degrees of proteins are different, the functional coefficient variation between the proteins is tiny small. Therefore, the functional difference between the proteins may be negligible and they share the same term. This just explains why some of the interacting proteins with different degrees share the same term. According to the results and analysis, the coefficient variation defined by GoDIN is effective to measure the functional difference between interacting proteins. The functional coefficient variation between interacting proteins can be considered as a positive clue to predict protein function.

### Comparison with the related methods

**Fig. 5:**
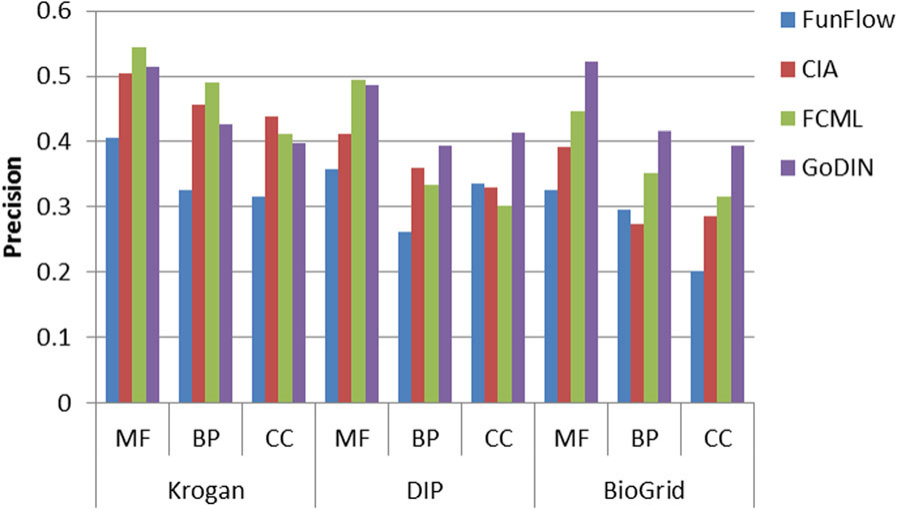
Comparison of precision of the related methods

**Fig. 6:**
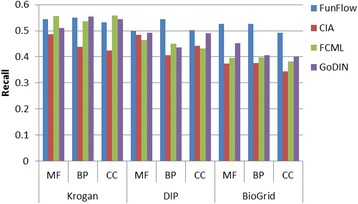
Comparison of recall of the related methods

To test the performances of GoDIN, we take FunFlow [7], CIA [17], FCML [18] as comparing methods. These comparing methods have been discussed in the introduction. They are three typical methods of predicting protein function based on PPI network and GO context respectively. The comparisons are performed on Krogan, DIP and BioGRID from three annotation aspects: molecular function (MF), biological process (BP) and cellular component (CC) respectively. Figures 4, 5 and 6 show the precision, recall and F-measure of these methods on different networks and annotation aspects.

As shown in Fig. 5, the precision of GoDIN is comparable to the best methods: CIA and FCML on Krogan. Meanwhile, GoDIN shows better precision than the other methods on DIP and BioGRID. FunFlow performs better than the others on DIP but it shows the lower precision than other methods on Krogan and BioGRID. In GoDIN, the functional differences of interacting proteins are considered and the differences of terms are used to demonstrate the functional differences during predicting protein function. This is why GoDIN shows better performances than the others in term of the precision. The functional relationships of terms are also considered thoroughly in CIA and FCML, but they pay no attention to the functional differences of interacting proteins. FunFlow ignores the functional relationships of terms in the process of predicting protein function so that it performs not as well as the others.

In addition, it is also found that all of the methods show relatively low accuracy. This may be due to two issues: (1) the large number of GO terms; (2) the dependency of GO terms. The influence of the above issues will be more obvious while the proteins are annotated by more terms. This would be a place to start the future study.

As displayed in Fig. 6, FunFlow shows the best recall on almost all of the networks while GoDIN performs better on most of the networks and annotation aspects than FCML and CIA. The performances of CIA are not better than those of FCML. This may be attributed to global characteristics and local characteristics of PPI network. Specifically, CIA only takes local characteristics of PPI network into consideration in predicting protein functions while the other methods consider both global and local characteristics of PPI network. This may be the reasons why the recall of CIA is lower than those of the other methods. Besides, some proteins in the datasets are annotated by shallow terms, and the misjudgments on these proteins have obvious negative impact on the recall. This would be a place to start our future study.

**Fig. 7:**
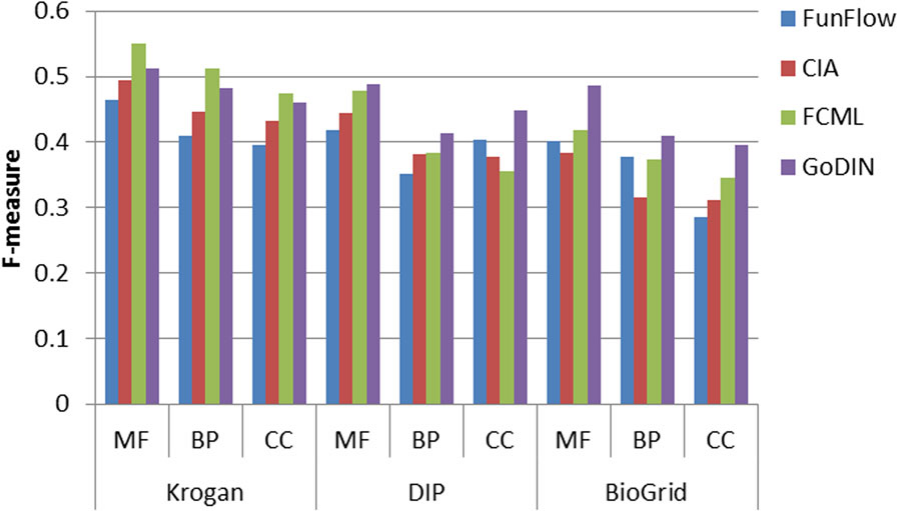
Comparison of F-measure of the related methods

As shown in Fig. 7, GoDIN performs almost as well as the best method FCML on Krogan while shows the best F-measure on DIP and BioGRID. FunFlow performs not better than the others on all of the networks. Overall, GoDIN shows better performances than the three methods in terms of metrics: precision, recall and F-measures when they are applied to predict protein functions.

## Conclusions

Predicting protein function based on PPI network is a hotspot of biological research in recent years. In this paper, the functional relationship between interacting proteins is studied and a novel method of protein function prediction is proposed based on the relationship. To validate the effectiveness of the method, a series of analysis and experiments are performed on the three high reliable networks from the different annotation aspects. The results suggest that: (1) interacting proteins are not equal in the PPI network, and their function may be same or similar, or just related; (2) functional difference between interacting proteins can be measured by their degrees in the PPI network; (3) functional relationship between interacting proteins can be expressed by semantic relationship between GO term and its relatives; (4) compared with the other concerned methods, GoDIN has high precision and f-measure and it is effective on predicting protein function.
